# The Impacts of Different Expansion Modes on Performance of Small Solar Energy Firms: Perspectives of Absorptive Capacity

**DOI:** 10.1155/2013/365089

**Published:** 2013-12-23

**Authors:** Hsing Hung Chen, Tao Shen, Xin-long Xu, Chao Ma

**Affiliations:** ^1^Faculty of Management and Administration, Macau University of Science and Technology, Taipa, Macau; ^2^Department of General Education, Macau University of Science and Technology, Taipa, Macau

## Abstract

The characteristics of firm's expansion by differentiated products and diversified products are quite different. However, the study employing absorptive capacity to examine the impacts of different modes of expansion on performance of small solar energy firms has never been discussed before. Then, a conceptual model to analyze the tension between strategies and corporate performance is proposed to filling the vacancy. After practical investigation, the results show that stronger organizational institutions help small solar energy firms expanded by differentiated products increase consistency between strategies and corporate performance; oppositely, stronger working attitudes with weak management controls help small solar energy firms expanded by diversified products reduce variance between strategies and corporate performance.

## 1. Introduction

The absorptive capacity, firm's capabilities to learn and absorb new knowledge, is seen as central to the performance of firms [1, 2]. The absorptive capacity is affected by organizational institutions [3] except for technological change and market needs [4]. Working attitudes significantly impact on corporate performance [5]. In addition, many researches have also shown that the performance of firms has substantially been impacted by their expansion modes [6]. Regarding the mode of expansion, small solar energy firms may expand their business by entering into new diversified products (by evolutionary and radical innovation) or by entering into new differentiated products (by revolutionary and incremental innovation). The characteristics of different expansion modes for firms are quite different [4].

The study employing absorptive capacity to examine the impacts of different modes of expansion on performance of small solar energy firms has never been discussed before. For the purpose of filling the vacancy, the paper builds on past research by suggesting that characteristics of different modes of expansion will impact on the firm's absorptive capacity, which in turn can determine performance of firms [7, 8]. Therefore, the relationships between modes of expansion and performance of firms should be mediated by absorptive capacity. To propose the assumptions, the study integrates absorptive capacity from two complementary theoretical perspectives. The first one is the institutional perspective of the firm. Many studies assert that the greater the normative embedded organization, the more likely that the organization will be revolutionary and convergent rather than evolutionary and radical [9]. The context of constitutions has important implications for access and transfer of knowledge. Therefore, the paper proposes that if a firm, adopting a set of formal institutions, restricts both distribution and access to knowledge sources, then these institutions would worsen financial performance in the long run. The second perspective is the working attitude of the employee. Many studies assert that most members in a dynamic organization (a more learning and evolutionary environment) used to show stronger favorable attitudes [5, 10]. The context of working attitude has also important implications for absorption and creation of knowledge. Therefore, the paper proposes that if a member of firms, possessing stronger working attitudes, portends both distribution and access to knowledge sources, then these attitudes would improve financial performance in the long run. Then, in order to solve the aforementioned proposition and find suitable corresponding operations management in different modes of expansion, the paper proposes a conceptual model to analyze the tension between product strategies and their strategy implementation [11, 12]. The results show that stronger organizational institutions help small solar energy firms expanded by differentiated products increase consistency between strategic targets and corporate performance; oppositely, stronger working attitudes with weak management controls help small solar energy firms expanded by diversified products reduce variance between strategic targets and corporate performance.

The rest of the paper is organized as follows. Critical characteristics of collaborating networks are studied in Section 2. A conceptual model and some hypothesized assumptions are proposed in Section 3. Data collection and analysis are presented in Section 4. Discussions and conclusions are addressed in the last sections.

## 2. Characteristics of Different Collaborating Networks

A fundamental process in industries and a source of competitive advantage are the introduction of new business (or new products) to the market [13]. Companies need to have successful new business (or new products) to confront fast changing technologies, shortening product life cycles, and increased global competition [14, 15]. Developing new business with network collaboration has become the main trend in the industry due to its advantages of knowledge accumulation, powerful competency, resources utilization, core technologies, organizational learning, social capitals inside a network, and an innovative environment for new business [16]. There are several challenges to technology-based producers with regard to choosing appropriate business strategies such as specialization, diversification, or scale increase, and meeting product quality, safety, sustainability, and customer satisfaction [17]. In face of these challenges, policy maker and the scientific literature now focus attention on entrepreneurship development [18]. A firm's production decision outcome marks two crucial production features, namely, technical and scale efficiency. Technical efficiency reflects the entrepreneurial ability to combine resources, that is, to produce maximum output given the bundle of available inputs and the technological characteristics of the firm. Technical efficiency is a paramount factor determined by a wide range of entrepreneurial and firm characteristics ranging from human capital quality and learning by doing abilities to research and development, innovation, and network development supporting a firm's expansion by diversified products. Scale efficiency reflects the ability to determine the optimum size of resources, that is, to decide on the size of the firm or, in other words, to choose the scale of production that will attain the produced level. Scale efficiency also is a factor reflecting a wide range of expansion modes such as future demand, the scale of economic operations, and differentiated expansion. Traditionally, developing new business by differentiated products is believed to bring merits such as lower cost, higher quality, tighter schedule, higher investment return and but may have risks such as vague market information, higher structure cost, lock-in technologies, and uncertain market demands [19]. Oppositely, developing new products by diversified products may result in advantages such as competency leveraging, capabilities transferring, knowledge flowing, information sharing, and multiple products, being exposed to dangers like cultural difference, over-estimated markets, and bureaucratic cost [19].

Many past works on developing new products were carried out from the point of view of external resource acquirers or value providers, based on the congenital and acquired characteristics of firms, external environment, and the interaction of individuals and opportunities. In addition to basic characteristics of a firm, the CSFs for developing new products are related to the acquisition of resources, especially the strategies that connect with the external social network [20]. Interpersonal network and strategic alliances among firms belong to a static network, and their characteristics are passive, releasing, adaptive, stable, systematic, and incremental [21, 22]. On the other hand, a less-focused point of view but with increasing importance is the external resource mobilization and value-creation organization. This stresses on the emergency process about how a focused firm can achieve new products through motivating others and influencing external environment. Such kind of research bases on the point of view of social construction and believes that a firm in a complicated network can find its own position and obtain its suitable developing condition through interaction, value creation, identification, and cooperation. Interpersonal network and strategic alliances among firms belong to a dynamic network, and their characteristics are dynamic, flexible, enabling, inside out, radical, collective, virtual, and program-oriented [21, 22].

Both of the above models focus on the relationship between external commercial model evolution and value creation, and this indicates the importance of a firm's positioning valuation. In other words, the character of a firm or its positioning, not only reflects customers' basic values, but also relates to the realization of the value of network style. To be more competitive in global competition, a firm's positioning should shift from value provider to value creator in an industrial value chain. Wheatley and Kellner-Rogers indicate that a dynamic network first needs to establish a clear and consistent organization identity, including vision, objectives, and values, in order to explain the beliefs and intention of the network in current environment. In addition, a focused firm, in order to create new products, needs to integrate the knowledge and abilities of external members, promote the standardization and systemization of knowledge structure and system, solve information asymmetry to promote knowledge flow, and provide professional service to increase customer knowledge [23]. The influential power of the focused firm comes from dynamic knowledge of the commercial and industrial networks; that is, a sharp cognition and reception of the discrepancies and potentials among systems can promote the evolution of network dynamics through meaning management or explanation building [24]. Under this kind of dynamic network, most members are self-motivated and energized with autonomy and involvement. Thus, the more dynamic the embedded network is, the stronger the favorable working attitudes of most members become. Finally, the characteristics of static and dynamic networks are listed in Table 1.

## 3. A Proposed Conceptual Model and Some Hypothesized Assumptions

### 3.1. The Proposed Conceptual Model

In order to examine the impacts of different expansion modes on the performance of small solar energy firms, a conceptual model to analyze the tension between strategies and corporate performance is proposed as follows:

Though Kaplan and Norton (1992) stressed that balanced scorecard (BSC) is a strategic management tool that transforms strategy into practice, many researchers held that BSC cannot feedback real operation results to strategic level, not to say to affect the establishment of strategies [11, 25, 26]. Besides, some scholars pointed out that many evaluation results of BSC are irrelevant to the success of strategies, and thus, the method may cause distortions [27]. There is the distortion between strategy formulation and strategy implementation [12]. Then, the distortion may be divided into (1) strategic level: irrelevance between strategic objectives and strategically selected projects; (2) executive level: inconsistency between corporate performance and strategically selected projects [11, 12]. A complete strategic framework should be able to handle long-term and short-term strategies, strategy formation and execution, diagnosis control, interacted control, and strategic control all together. Then, to solve the first problem, we adopt path analysis to find the relationship between CSFs of new products and strategic objectives. To solve the second problem, we adopt path analysis to find out the relationship between CSFs of new products and performance of firms.

After simplifying CSFs of new products by factor analysis, path analysis is then carried out diachronically to determine the relationships between strategic objectives and extracted CSFs and between extracted CSFs and corporate performance.

### 3.2. The Proposed Hypotheses

*Hypothesis (a).* Small solar energy firms expanded by diversified products (by evolutionary and radical innovation) have the better executive capabilities for strategic targets at strategic level.

Basically, small solar energy firms expanded by differentiated products are external resource acquirers or value providers based on the interaction of individuals and opportunities. Thus, most members have weaker common visions and beliefs [4], originated from the characteristics of static networks listed in Table 1. Favorable working attitudes such as organizational autonomy and willingness to take risks may be weaker [10]. Oppositely, small solar energy firms expanded by diversified products need to actively integrate the abilities of external members, solve information asymmetry to promote knowledge flow, and provide professional service to increase customer knowledge. Then, most members have stronger visions and beliefs [4, 10], originated from the characteristics of dynamic networks listed in Table 1. Then, favorable working attitudes such as innovativeness and proactive assertiveness may be stronger [28]. Thus, the relationships between strategic objectives and extracted CSFs for organizations expanded by diversified products are stronger than those for organizations expanded by differentiated products. This indicates that the executive capability for strategic targets at strategic level favors organizations expanded by diversified products.

*Hypothesis (b).* Small solar energy firms expanded by differentiated products (by revolutionary and incremental innovation) have the better executive capabilities for operational targets at executive level.

Basically, small solar energy firms expanded by differentiated products put their first priority on perusing efficiency (such as lower cost and modular services) and effectiveness (such as higher quality and tighter schedule). Developing new products by efficiency and effectiveness may result in lower capacity for action and reformative commitment [9]. This means that firms face more competitive commitment. Most members of firms possess stronger organizational institutions for competitive environments [29]. Since they are in pursuit of efficiency and effectiveness, tighter management controls should be applied in the organizations [30]. Oppositely, small technology-based organizations expanded by diversified products have the characteristics of transferring capabilities, leveraging competencies, flexible schedule, radical innovation, and multiple products. Developing new products by flexibility and leveraging may result in higher capacity for action and reformative commitment [30]. Most members of organizations own weak organizational institutions for creating innovative environments [29]. Thus, looser management controls should be more suitable to organizations with evolutionary and radical environments [9, 10]. Thus, the relationships between extracted CSFs and performance evaluation for organizations expanded by differentiated products are stronger than those for organizations expanded by diversified products. This indicates that the executive capability for operational targets at executive level favors organizations expanded by differentiated products.

*Hypothesis (c).* Small firms expanded by differentiated products have more advantages on efficiency and effectiveness.

Developing new products by differentiated products may bring merits such as lower cost, higher quality, tighter schedule, and better investment return [19]. Organizations have stronger tendency to maximize their short-term profits since they may produce higher efficiency and effectiveness.

*Hypothesis (d).* Small firms expanded by diversified products have more merits on organizational learning.

Developing new products by diversified products may result in advantages such as competency leveraging, capabilities transferring, knowledge flowing, information sharing, and bundling services [19]. Organizations have stronger tendency to maximize their long-term profits since they may produce higher organizational learning and then make huge profit in the long run.

*Hypothesis (e).* The consistent relationships between strategic objectives and corporate performance for small solar energy firms expanded by differentiated products are stronger than those by diversified products.

The strategies for organizations expanded by differentiated products are short-term, significant (value providers), explicit (efficiency and effectiveness), and exploratory (incremental innovation). However, the strategies for organizations expanded by diversified products are long-term, obscure (value creators), implicit (organizational learning), and exploitative (radical innovation).

## 4. Data Collection and Analysis

### 4.1. Sample Selection

In order to employ absorptive capacity to mediate the impacts of different expansion modes on corporate performance, the paper selected small solar energy firms in different sectors as our targeted firms. Totally, 606 Chinese small solar energy firms located in the province of Shanghai, Shandong, Jiangsu, and Zhejiang had been selected as our samples. After going through the extensive literature review and discussing with 23 managers working in 10 firms experiencing successful expansion, we included four strategic objects, 45 possible CSFs of new products development, and four performance evaluation indices in the questionnaire. Totally, there were 53 questions in the questionnaire. The purpose of the questionnaire was to examine the importance of each factor to a firm's long-term competitive performance. The closed questionnaire was evaluated by Likert scale, with 1 standing for least importance and satisfaction and 5 for greatest importance and satisfaction. Questionnaires were, respectively, distributed to bosses, managers, and supervisors involved in the 606 targeted firms in the end of 2012. In addition, all invited firms have had successful expansion either by differentiated or diversified products for more than two years. Those firms have utilized proper management controls to design their strategies and monitor their performance. In order to ensure validity of survey data, normal post mails supplemented with direct communication were used to track individuals. A total of 5,000 questionnaires were sent, and 984 questionnaires were received, with a returning rate of 19.68%. Statistical analysis of the returned questionnaires shows that both reliability (Cronbach *α*) and validity (average-variance extracted; AVE) coefficients of questionnaires in different stages are above 0.7, and it means that reliability and validity of returned questionnaires are acceptable, as shown in Table 2. Finally, the data were analyzed using a *t*-test procedure; there is no significant difference (*P* < 0.05) between the interview and mailed responses. Since some variables may influence the results, variables such as age, gender, areas, and level of education were examined. However, the results did not show any significant difference.

### 4.2. Factor Analysis

Regarding 45 possible CSFs of new products development, 13 CSFs with eigenvalues larger than 1.00 were extracted as common factor dimensions through factor analysis and varimax rotation method by SPSS software 19.0 [31–33]. Kaiser-Meyer-Olkin (KMO) statistics were used to measure sampling adequacy, that was, if data were likely to factor well. Since the KMO statistic was 0.723, a value greater than the satisfactory value of 0.5, it was appropriate to proceed with factor analysis. In addition, Bartlett's test of sphericity tests the null hypothesis that the variables in the correlation matrix are uncorrelated. Since the observed significance level was 0.000, it was small enough to reject the hypothesis. This also suggested that a factor analysis for the data could proceed, and eigenvalues, variance, and cumulative variance of the 13 selected CSFs could explain 78.2% of the variance in the original data sets. For the naming of extracted factors, this research chose a loading factor in each dimension larger than 0.40 as a reference for the name and used a name that represented the aggregates of the observed factors. The extracted factors, dimension one through thirteen, were listed as follows: competitive advantage, human resources, market potential, technological characteristics, technology accumulation, integrated resources, surviving capabilities, market share, services capabilities, return of investment, organizational learning, customer satisfaction, and technological improvement.

### 4.3. Cluster Analysis

In this part, different characteristics of networks listed in Table 1 are compared regarding firms expanded by diversified products and differentiated products. These comparisons involve MANOVA test with Bonferroni posthoc pairwise comparison test. This will help to verify characteristics of different modes of expansion.

The questions about the modes of expansion are asked using a 5-point Likert scale inquiring how important each characteristic (such as radical or systematic) is for the respondents with the scale ranging from 1 = extremely unimportant to 5 = extremely important.  Table 3 shows that they significantly differ at 5% level, except for adaptive, inside-out, and program-oriented characteristics and enabling energy. First, it shows that the most important characteristics for firms expanded by differentiated products are modular product, short-term profit, systematic integration, and incremental and passive innovation. The results make sense since the mode of expansion by differentiated products is in pursuit of effectiveness and efficiency. Second, it shows that the most important characteristics for organizations expanded by diversified products are dynamic and radical innovation and organizations created by value. The results make sense since characteristics of diversified expansion are in pursuit of learning and the subsequent radical innovation. Finally, the answered questionnaires were categorized into two expansion modes for subsequent investigation.

### 4.4. Path Analysis

Here, path analysis was applied to examine the relationship between the 4 strategic objectives and the 13 extracted CSFs, and then the relationship between the 13 extracted CSFs and the 4 performance evaluation indices. The two path analysis models were integrated last to form a complete evaluation model. There was a time sequence among the stages.

#### 4.4.1. Expansion by Differentiated Products (from 538 Questionnaires)

(a) Path analysis model (I): path analysis of the 4 strategic objectives and the 13 extracted CSFs as carried out. The results were shown in Table 4.

(b) Path analysis model (II): path analysis of the 13 extracted CSFs and the 4 performance evaluation indices as carried out. The results were shown in Table 5.

(c) Cascading path analysis models (I) and (II) together: by integrating the results of path analysis models (I) and (II), we deduced that there were relationships among strategic objectives, extracted CSFs, and performance evaluation and that the relationships were relevant according to time sequence.

#### 4.4.2. Expansion by Diversified Products (from 446 Questionnaires)

The same path analyses were carried out as in Section 4.4.1 for diversified expansion, and the results were shown in Tables 6 and 7.

## 5. Analysis and Discussion

Generally speaking, the path coefficients between strategic objectives and extracted CSFs for organizations expanded by differentiated products are smaller than those for organizations expanded by diversified products, and organizations expanded by differentiated products have two fewer extracted CSFs, that is, lack of market share and services capabilities, as compared in Tables 4 and 6. Basically, organizations expanded by diversified products need to actively integrate the abilities of external members, solve information asymmetry to promote knowledge flow, and provide professional service to increase customer knowledge. Members usually possess stronger visions and beliefs, and favorable working attitudes are resulted. Since the relationships between strategic objectives and extracted CSFs indicate the executive capabilities for strategic targets, organizations by diversified expansion have a better strategic capability at strategic level. Then, hypothesis (a) is proved to be held. Oppositely, by comparing Tables 5 and 7, the path coefficients between extracted CSFs and performance evaluation for firms expanded by diversified products are smaller than those expanded by differentiated products, and the relationships are rather different. Basically, organizations expanded by differentiated products put their first priority on perusing efficiency and effectiveness. Most members of firms possess stronger organizational institutions for competitive environments. Since the relationships between extracted CSFs and performance evaluation indicate the executive capability for operational targets, organizations expanded by differentiated products have a better executive capability at operational targets. Then, hypothesis (b) is proved to be held. Since organizations expanded by differentiated products are in pursuit of efficiency and effectiveness, tighter management controls are recommended for them.

When cascading the data in Tables 4 and 5, the same dimensions found in both Tables 4 and 5 focus on effective and efficient perspectives. The characteristics of passive, releasing, adaptive, stable, systematic, incremental, and system-oriented network summarized in Table 1 will create nonpermeable, normative embedded, tightly coupled, and reformative organizational and institutional environments and then will result in characteristics of lower cost, higher quality, tighter schedule, and better investment return. It means that organizations expanded by differentiated products are in pursuit of efficiency and effectiveness. Then, hypothesis (c) is proved to be held. Oppositely, when cascading the data in Tables 6 and 7, the same dimensions found in both Tables 6 and 7 focus on learning and innovative perspectives. The characteristics of dynamic, flexible, enabling, inside out, radical, collective, and program-oriented summarized in Table 1 will build permeable, nonnormative embedded, loosely coupled, and competitive organizational and institutional environments and will subsequently bring competency leveraging, capabilities transferring, information sharing, knowledge flowing, and multiple products. It means that organizations expanded by diversified products stress on organizational learning. Additionally, there is a positive path coefficient among technological capabilities (from strategic objectives), organizational learning (from extracted CSFs), and innovation perspectives (from performance evaluation) for organizations expanded by diversified products. Therefore, hypothesis (d) is proved to be held. Finally, the consistent relationships between strategic targets and corporate performance are stronger for organizations expanded by differentiated products than those for organizations expanded by diversified products, by comparing Tables 4 and 5 with Tables 6 and 7. Thus, hypothesis (e) is proved to be held.

**Figure 1:**
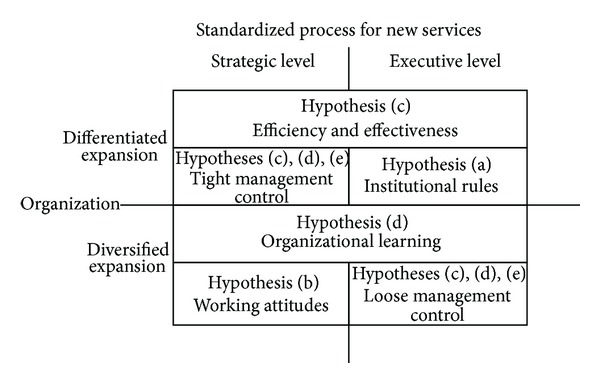
The summarized results from hypotheses.

From Figure 1, it is concluded that stronger favorable working attitudes with weak management controls help small solar energy firms expanded by diversified products reduce variance between strategic targets and corporate performance. Oppositely, stronger organizational institutions help small solar energy firms expanded by differentiated products increase consistency between strategic targets and corporate performance.

## 6. Conclusions

Institutional theory shows how organizational behaviors respond not solely to market and technological pressures, but also to institutional pressure. The paper considers that working attitudes like organizational autonomy, risk taking, resource mobilization, and innovativeness also play an important role to corporate performance. This reinforces the theoretical integrity of organizational behaviors. In addition, the absorptive capacity is affected by organizational institutions and working attitudes and seen as central to the performance of firms. The study first employs absorptive capacity to examine different modes of expansion on the performance of small solar energy firms. Then, suitable management controls can be applied accordingly.

Traditionally, due to systemic failures, both strategic objectives and corporate performance have experienced constraints in effecting operations and then establishing the necessary relationships to reduce the gaps is desired. Then, the paper suggested that small solar energy firms should continuously examine whether strategically selected projects depart from strategic objectives, that is, strategically selected projects should conform to dynamic market demand and technological development trend. At the same time, a firm should also continuously examine whether performance evaluation results depart from expected performance objectives. To solve the first problem, the paper adopts path analysis to monitor relationships between extracted CSFs and strategic objectives. To solve the second problem, the paper applies path analysis to diagnose the relationships between extracted CSFs and project performance. With such a design, to solve the two problems, inconsistency between strategic objectives and final performances may be reduced. Finally, the paper concludes the following improvements with a number of policy recommendations after such a design is applied.

Small solar energy firms often experience difficulties in defining strategic, organizational, and technological deficiencies in their efforts to express clear demands so that they get a product that meets their requirements. Conversely, small technology-based firms also have to be responsive to customer needs, that is, they have to be demand driven. Cognitive distance between the different actors involved may cause coordination and learning problems during expansion. Then, inconsistencies raised by such a systematic design should be checked by absorptive capacity to timely reduce the gap.

Small solar energy firms expanded by diversified products require more initiatives from entrepreneurship. This calls for competences with regard to knowledge and information acquisition and learning for innovation, that is, sufficient absorptive capacity. Such competences are often lacking in small firms; that is, there is an information gap. This complicates the search for suitable cooperation partners, especially with regard to accessing “weak tie” networks that can offer opportunities. Then, inconsistencies raised by such a then, inconsistencies raised by such a systematic design should be checked by absorptive capacity to timely reduce the information gap.

Small solar energy firms expanded by differentiated products often lack capabilities of integrating resources such as the mode to coordinate with other actors. In the increasingly heterogeneous market for small solar energy firms, services failures such as asymmetry information, inefficient operations, and poor identification of service value can be discerned. This implies difficulties in ex ante evaluation; that is, there exists a managerial gap. This complicates the selection of suitable coordination modes, especially with regard to accessing “strong tie” networks that can offer efficiency. Then, inconsistencies raised by such a systematic design should be checked by absorptive capacity to timely reduce the managerial gap.

## Figures and Tables

**Table 1:** The characteristics of static and dynamic collaborating networks.

Static network	Dynamic network
Efficiency	Effectiveness
Releasing energy	Enabling energy
Adaptive	Collecting
Incremental innovation	Radical innovation
Modular product	Multiple product
Passive	Active
Outside-in	Inside-out
Static	Dynamic
Satisfying the market need	Creating the market need
System-oriented	Program-oriented
Maximizing short-term profit	Maximizing long-term profit
Value created by organization	Organization created by value

**Table 2:** Reliability and validity coefficients at different stages of questionnaires.

Research stage	Aspect of variables	Questions	AVE	Cronbach *α*
Strategy stage	4 strategic objectives	4	0.8217	0.7942
New product development	45 critical success factors	45	0.7631	0.8021
Evaluation stage	4 modified BSC	4	0.7532	0.7858

**Table 3:** Characteristics of networks under different expansion modes.

Different modes of expansion and Different characteristics of networks	Differentiated products	Diversified products	*F* (or *K*)
Dynamic characteristics (cluster mean)	1.86^a^ (2)^b^	3.65 (1)	11.23^c^ *P* < 0.043
Passive innovation (cluster mean)	2.89 (2)	1.91 (1)	15.26 *P* < 0.032
Incremental innovation (cluster mean)	3.48 (2)	2.48 (1)	10.31 *P* < 0.045
Modular product (cluster mean)	2.89 (2)	1.43 (1)	10.24 *P* < 0.046
Adaptive characteristics (cluster mean)	3.36	2.96	2.62 *P* < 0.0125
Systematic-oriented integration (cluster mean)	3.25 (2)	2.14 (1)	8.36 *P* < 0.072
Radical innovation (cluster mean)	1.34 (2)	4.13 (1)	13.31 P < 0.034
Inside-out characteristics (cluster mean)	1.83	3.51	5.22 P < 0.096
Program-oriented characteristics (cluster mean)	1.28	2.21	4.25 P < 0.099
Short-term profit (cluster mean)	3.93 (2)	2.27 (1)	13.14 P < 0.035
Enabling energy (cluster mean)	1.97	2.34	3.98 P < 0.0117
Organization created by value (cluster mean)	1.89 (2)	3.29 (1)	9.36 *P* < 0.053

Note: ^a^mean based on 5-point Likert scale comparing the data collected in the end of 2012.

Note: ^b^numbers in parentheses indicate the cluster groups from which this cluster is significantly different at α = 0.05 according to the Bonferroni, posthoc pairwise comparison procedures.

Note: ^c^ *F* and corresponding *P* values based on MANOVA test.

**Table 4:** Path analysis between 4 strategic objectives and 13 extracted CSFs. (Small technology-based firms expended by differentiated products.)

Dependent variables	Independent variables	Absolute and standardized path coefficients	*P* value	Adjusted *R* ^2^
Competitive advantage	Technological capability	0.314	0.003***	0.305
Market potential	Market capability	0.156	0.031**	0.212
Human resources	Organizational relationship capability	0.108	0.075*	0.105
Integrated resources	Integrating capability	0.193	0.037**	0.184

*P < 0.1; **P < 0.05; ***P < 0.01.

**Table 5:** Path analysis between 13 extracted CSFs and 4 performance perspectives. (Small technology-based firms expended by differentiated products.)

Dependent variables	Independent variables	Absolute and standardized path coefficients	*P* value	Adjusted *R* ^2^
Financial perspective	Market potential	0.355	0.005***	0.318
	Market share	0.252	0.015**	0.318
	Return of investment	0.136	0.029**	0.318
Customer perspective	Customer satisfaction	0.114	0.048**	0.225
	Surviving capabilities	0.112	0.061*	0.225
Internal business perspective	Integrated resources	0.263	0.014**	0.369
	Surviving capabilities	0.391	0.004***	0.369
	Services capabilities	0.156	0.052*	0.369

*P < 0.1; **P < 0.05; ***P < 0.01.

**Table 6:** Path analysis between 4 strategic objectives and 13 extracted CSFs. (Small technology-based firms expended by diversified products.)

Dependent variables	Independent variables	Absolute and standardized path coefficients	*P* value	Adjusted *R* ^2^
Competitive advantage	Technological capability	0.415	0.001***	0.381
Market potential	Market capability	0.126	0.062*	0.142
Market share	Market capability	0.168	0.041**	0.146
Organizational learning	Organizational relationship capability	0.302	0.004***	0.271
Integrated resources	Integrating capability	0.267	0.025**	0.335
Services capabilities	Integrating capability	0.265	0.011**	0.273

*P < 0.1; **P < 0.05; ***P < 0.01.

**Table 7:** Path analysis between 13 extracted CSFs and 4 performance perspectives. (Small technology-based firms expended by diversified products.)

Dependent variables	Independent variables	Absolute and standardized path coefficients	*P* value	Adjusted *R* ^2^
Financial perspective	Competitive advantage	0.202	0.048**	0.211
	Market share	0.165	0.062*	0.212
Customer	Customer satisfaction	0.113	0.082*	0.117
Innovation	Organizational learning	0.267	0.031**	0.246

*P < 0.1; **P < 0.05; ***P < 0.01.
